# Efficient Single Nucleotide Polymorphism Marker-Assisted Selection to Fusarium Wilt in Chickpea

**DOI:** 10.3390/plants13030436

**Published:** 2024-02-01

**Authors:** Patricia Castro, Cristina Caballo, Alejandro Carmona, Teresa Millan, Juan Gil, José V. Die, Inmaculada Izquierdo, Josefa Rubio

**Affiliations:** 1 ETSIAM-Dpto. Genética, Universidad de Córdoba, Campus de Rabanales, 14071 Córdoba, Spain; b62cajia@uco.es (A.C.); teresa.millan@uco.es (T.M.); jose.die@uco.es (J.V.D.); 2 Área de Mejora Vegetal y Biotecnología, Instituto Andaluz de Investigación y Formación Agraria, Pesquera, Alimentaria y de la Producción Ecológica (IFAPA), 14080 Córdoba, Spainjosefam.rubio@juntadeandalucia.es (J.R.); 3 SCA Campo de Tejada, 21870 Escacena del Campo, Huelva, Spain

**Keywords:** breeding, chickpea, fusarium wilt, marker assisted selection, SNPs

## Abstract

Fusarium wilt is one of the most destructive chickpea diseases worldwide. Race 5 (Foc5) is the most harmful in the Mediterranean basin. The primary objective of this study is to validate a block of six SNP markers previously mapped in Ca2 in a diverse panel of cultivars, advanced and inbred lines phenotyped for resistance to fusarium wilt. Additionally, we aim to assess the effectiveness of using these markers in the selection of resistant Foc5 lines in an ongoing breeding program. The results showed a 100% coincidence between phenotype and expected haplotype in plant material evaluated for Foc5. We also analyzed 67 inbred lines previously phenotyped by different authors for fusarium wilt reaction, though the specific race was not specified. In these accessions, 65.8% of the analyzed lines exhibited complete correspondence between the phenotype and haplotype. Our results suggest that in early generations it is possible to select resistant materials with reliability, leading to the removal of a significant number of lines, thereby reducing costs and facilitating the handling of materials for additional trait evaluations. Functional annotation of genes delimited by the SNP block revealed several genes in the “response to stimulus” category with potential roles in the resistance reaction.

## 1. Introduction

Fusarium wilt in chickpea, caused by the soilborne fungus *Fusarium oxysporum* f. sp. *ciceris* (Foc), is one of the most devastating chickpea diseases worldwide. The use of resistant cultivars is widely acknowledged as the most practical and environmentally friendly solution for managing this disease. Consequently, the selection of Foc-resistant genotypes is a key objective in chickpea breeding programs globally. The identification of eight physiological races of the fungus, based on cultivar specificity, hampers the effectiveness of developing resistant cultivars. Hence, it is advisable to develop multi-race-resistant varieties. On the other hand, the genetic inheritance of resistance reaction has primarily been described as oligogenic, simplifying the breeding process. Mendelian genetic studies have determined that, depending on the race, a maximum of three genes control resistance reactions for Foc [1,2,3]. Resistance to race 5 (Foc5), an important race in the Mediterranean basin and the focus of this study, is governed by a single recessive gene [4]. Analysis of molecular markers in RIL (Recombinant Inbred Line) populations has allowed the mapping of Foc resistance genes in genetic maps, emphasizing linkage group 2 (LG2) or chromosome 2 (Ca2), where there is a cluster of resistance genes to several races [1,5]. Microsatellite markers located in LG2/Ca2, such as GA16, TA59, TA96, TA194, TA110, TAA60, TR19, TR29, TR31, and TS82 have been used for screening, validation, and development of fusarium wilt-resistant chickpea genotypes against prevalent races in India (1, 1A, 2, 3, and 4) [6,7]. Additionally, TA59, which is tightly linked to Foc5, has been used to develop near-isogenic lines (NILs) [8] and large-seed Spanish white-type cultivars resistant to Foc5 using Marker Assisted Backcrossing (MAB) [9].

Microsatellite markers can present challenges when analyzing materials with an unknown pedigree. In such cases, these markers have the potential to unveil new alleles without prior knowledge of their association with either resistance or susceptibility. The availability of chickpea whole-genome sequences [10,11] and previously established genetic maps facilitates the positioning and saturation of targeted genomic regions. Our research group performed fine mapping in LG2/Ca2 starting from TA59 as a reference marker [12]. In that study, the saturation of the genomic region was achieved using single nucleotide polymorphism (SNP) markers through a comparative analysis involving the reference genome CDC Frontier and re-sequencing data from genotypes WR315 (resistant to all Foc races) and ILC3279 (susceptible) and segregant plant materials (RILs and NILs). The targeted region, covering ~2 Mpb, included a block of six SNPs exhibiting distinct haplotypes associated with either Foc5-resistant or susceptible genotypes. These associations were identified based on different sources of resistance that included the set of differential lines described by Sharma et al. [13].

To determine the practical efficacy of the block of six SNP markers in breeding programs, it is highly desirable to genotype a larger set of lines with varied origins. Therefore, the primary objective of the present study is to validate the aforementioned markers in a diverse panel of cultivars, advanced and inbred lines that have been phenotyped for their resistance reaction to fusarium wilt. In addition, we aim to assess the effectiveness of using these markers in the selection of resistant Foc5 lines within the context of an ongoing breeding program. Finally, the ultimate goal is to provide some insights into the molecular identity and potential function of genes within the region of interest through functional annotation. Identifying specific genes associated with a particular trait within a narrowed-down genomic region not only deepens our understanding of gene function and the underlying genetic basis of the trait but also enables the development of diagnostic markers. These markers have the potential to significantly expedite breeding programs by streamlining selection processes with increased precision.

## 2. Results

### 2.1. Evaluation of Accessions and Advanced Lines

In this study, 61 accessions, including cultivars and advanced lines (Table 1), were genotyped to assess the alignment between phenotypes and the anticipated haplotype of the SNP block targeting the genomic region associated with Foc5. In all cases, there was a 100% coincidence between phenotype and expected haplotype (resistant: AAACAA; susceptible: GCGGGT). The three resistant and the four susceptible controls exhibited the expected haplotype associated with resistance or susceptibility, respectively, reinforcing the results obtained here.

We also analyzed 67 inbred lines previously identified by different authors as exhibiting resistant, susceptible, or intermediate reactions for fusarium wilt, though in most cases the specific race of Foc was not explicitly specified (Table 2). Among these lines, 38 were reported to have a clearly defined resistant (R) or susceptible (S) phenotype (ID: 62 to 99; n = 38). The result of the genotyping (using six SNPs) showed a concurrence between the phenotype and the expected haplotype in 25 of these lines. Specifically, 14 of them were resistant (ID: 62 to 75) and 11 were susceptible (ID: 76 to 86). In the remaining 13 lines, there was no correspondence between the observed phenotype and the expected haplotype. Eight lines, phenotyped as resistant (ID: 87 to 94), exhibited the susceptible haplotype, indicating that they may be resistant to other Foc races but not to Foc5. Meanwhile, two lines, also phenotyped as resistant (ID: 95 and 96), presented a haplotype that differed in one (SNP40) and two (SNP8 and SNP14) with the resistant patterns.

Additionally, two lines initially phenotyped as susceptible (ID: 97 and 98) displayed the resistant haplotype. The susceptible line ID: 99 was heterozygous for SNP8 and SNP30, showing the susceptible genotype for the rest of the haplotype. It is noteworthy that 65.8% of the analyzed lines exhibited complete correspondence between the phenotype and haplotype. This suggests that the SNPs are useful to select resistant plants for Foc5 but it could be risky to use them for other Foc races due to the possibility of losing resistant donors with different resistance mechanisms. Among the 29 inbred lines phenotyped with an intermediate reaction (I), 17 accessions presented the resistant haplotype for Foc5 (ID: 100 to 116), while 11 had the susceptible haplotype (ID: 117 to 127). Intriguingly, one accession (ID: 128, ICC5704) presented all the alleles corresponding to the susceptible haplotype, except for a single change in SNP40, where an A was present instead of a T (Table 2).

### 2.2. MAS Application

We analyzed 3634 F_2_ plants derived from multiple crosses between susceptible and resistant Foc5 genotypes (Table 3). In the year 2021, 498 F_2_ plants were genotyped using the complete SNP block. The SNP data fit well to a segregation ratio of 1:2:1 (129R:245H:124S; Chi-square = 0.23; *p* = 0.89), as expected for a single gene in an F_2_ generation. Notably, all individuals exhibited the anticipated SNP alleles, with no detection of any recombination event within the block. Consequently, in an attempt to streamline costs in our breeding program the following year, we decided to genotype the F_2_ plants using only the two SNPs located at the extremes of the block (SNP8 and SNP40). In 2022, a total of 1885 F_2_ plants were analyzed with these two SNPs, and the segregation also fit well to the expected 1:2:1 ratio (486R:969H:430S; Chi-square = 4.82; *p* = 0.09). This indicates that these two SNPs could be sufficient for selecting resistant plants in a breeding program. To confirm the effectiveness of using two SNPs instead of the entire SNP block for confidently selecting F_2_ plants with the expected haplotype, we compared the F_2_ segregation data from specific crosses (Table 3). For instance, the F_2_ population resulting from the cross between TK-18 and ILC187 was genotyped in both 2021 and 2022 using the complete SNP block and the two selected SNPs, respectively. In both instances, the obtained results aligned well with the expected 1:2:1 segregation (30R:75H:32S vs. 16R:36H:14S, and *p* = 0.52 vs. 0.72, respectively). Encouraged by these results, and as part of our continued initiative to minimize genotyping costs in our breeding program, we further attempted to reduce the number of SNPs to just one in the subsequent year. In 2023, we genotyped 1251 F_2_ using only SNP8, and the segregation observed was 314R:638H:299S, fitting a 1:2:1 ratio (Chi-square = 0.86; *p* = 0.65). These findings demonstrate that genotyping a single SNP of the SNP block reported by Caballo et al. [12] enables a cost reduction in a breeding program without compromising the efficiency of selecting resistant plants. On the other hand, reducing the number of markers to only one could make it difficult to detect recombinations between the SNP markers to break haplotypes for better mapping and identification of candidate genes. So, another way of cost reduction without reducing the number of markers could be to convert the SNP to KASP markers.

### 2.3. GO Analysis

**Figure 1:**
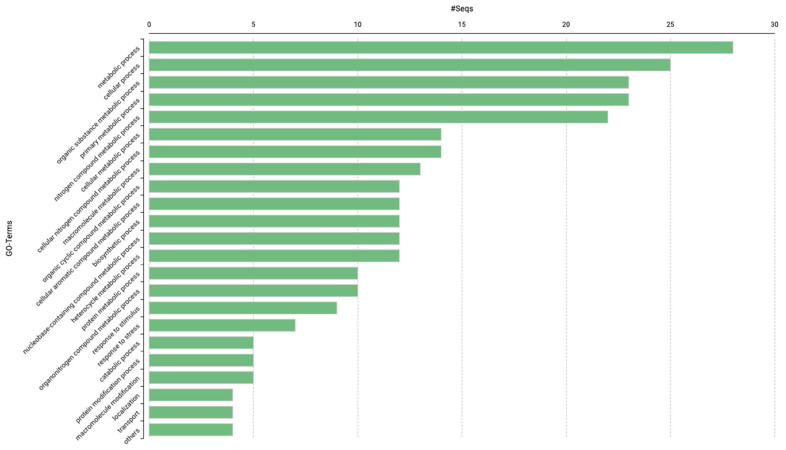
Gene ontology “biological process” category distribution of annotated genes in the genomic region delimited by the SNP markers.

To identify candidate genes that could be responsible for the control of Foc5 resistance, we analyzed the genomic region comprising the block of SNPs. That block spans approximately 1.52 Mb and contains 79 loci. After removing 11 entries described as “pseudogenes,” a set of 68 sequences was processed through the functional annotation pipeline, resulting in full annotation (Table S1). The distribution of GO terms and sequences across the “biological process” ontologies is shown in Figure 1. In the “biological process” ontology, the class “response to stimulus”, contains nine protein sequences. Notably, five of these sequences are associated with the category GO:0006950 “response to stress”. Among this group LOC101500060 and LOC105851626 encode an MLO-like protein, and an enhanced disease-resistance protein, respectively. Additionally, three other sequences are also classified within the “molecular function” ontology, including a detoxification protein (LOC101495941) and a CBL-interacting serine/threonine-protein kinase (LOC101511605). Finally, a pathogenesis-related protein (LOC101510320) from this class is categorized in the “cellular component” ontology (GO:0005576, extracellular region). 

## 3. Discussion

The extent of economic damage from fusarium wilt in chickpea depends on the successful development of resistant cultivars. Although classical breeding methods have, at times, demonstrated effectiveness in creating resistant materials, they also present difficulties, such as environmental influences, complexity, and time-consuming evaluation techniques. The integration of genomics into chickpea improvement is expected to simplify breeding for this biotic stress.

The use of marker loci closely linked to essential genes regulating features with economic relevance, such as disease resistance, can assist in more effective selection. Nowadays, SNP-based markers are considered the markers of choice for plant breeding programs. The preference is attributed to their abundance among individuals of the same species, genome-wide distribution, and cost-effectiveness flexibility [21].

The complete alignment between the expected haplotype of the SNP block and the observed phenotype in cultivars and advanced lines evaluated for Foc5 (Table 1) reveals the utility of these markers for more efficient resistance breeding against this race. The 61 chickpea accessions described in Table 1, which inherited resistance from WR315 and ICC81001 in their pedigree for Foc5, consistently exhibited the expected haplotype for both resistance and susceptibility upon genotyping with the SNP block. Remarkably, despite undergoing various recombinant events, this SNP block remains stable.

Additionally, a high percentage (65.8%) of the inbred lines, previously evaluated by other authors and characterized as clearly resistant or susceptible, exhibited a perfect match between their phenotype and the haplotype derived from the SNP block. Among these lines, only 13 showed discrepancies between phenotype and haplotype. One of the 13 accessions was evaluated for races 1 and 2 but the Foc race was not specified for the remaining lines. It should be noted that these evaluations took place in India where races 1, 1A, 2, 3, and 4 are prevalent in the fields. Therefore, it can be assumed that the lines were resistant to any of these races but not necessarily to Foc5. Importantly, it should be considered that the selected haplotype block was initially designed for Foc5 resistance, a race distributed in the Mediterranean basin. Hence, breeders should be cautious when selecting resistance for races other than Foc5 using this SNP block. For completely unknown plant materials, it would be generally recommended to perform the resistance test for these lines under their conditions, perform parallel molecular characterization, and then use the markers in the progenies. The effectiveness of selection with this SNP block in materials evaluated for different Foc races could be explained by the presence of a genomic region on chromosome 2 (LG2/Ca2), referred to as a “hotspot”, that harbors multiple resistance genes for Foc races [3,22,23]. The presence of differential lines—resistant to some races and susceptible to others—suggests the possibility of recombination in this region of chromosome 2.

The functional annotation of the region delimited by the SNP block revealed several genes in the “response to stimulus” category (GO:0050896) with potential roles in the resistance reaction. Thus, LOC101500060 encodes an MLO-like protein, LOC105851626, an enhanced disease resistance protein, LOC101495941, a detoxification protein, and LOC101510320, a pathogenesis-related protein. Another GO term of interest “signal transduction” (GO:0007165) was associated with LOC101511605, which encodes a CBL-interacting serine/threonine-protein kinase. This locus was suggested as a candidate gene with a significant role during the resistance reaction [12]. Interestingly, The SNP8 is located within the gene sequence of LOC101507025, a locus encoding a SABRE-like protein that was annotated in the category “membrane” (GO:0016020) in the cellular compartment ontology. Further investigations to fine mapping in this genomic region are necessary to precisely identify markers targeting resistance genes for different Foc races.

Concerning the lines phenotyped as intermediate (Table 2), nearly all of them, with the exception of one, exhibited either a resistant haplotype (60.7%) or a susceptible haplotype (39.3%). The classification of an intermediate phenotype raises questions, as different disease scoring scales are used to determine phenotypic resistance and susceptibility [13,23]. On the other hand, the lines phenotyped as intermediate were evaluated mainly in India, where race 5 has never been reported. Resistance to the races prevalent in India (1, 1A, 2, 3, and 4) is controlled by more than one gene (up to three depending on the race) [3]. Therefore, it could be possible that those lines carry only some of the genes controlling the resistance, leading to intermediate responses.

In conclusion, in this study, we initially genotyped the chickpea material using the Sequenom Mass ARRAY iPLEX Platform methodology [24] with excellent results, using a block of six SNPs. Subsequently, we applied this SNP block in a practical case study (MAS application), involving genotyping F_2_ plants derived from multiple crosses between those resistant and susceptible to Foc race 5 in the year 2021. However, in the subsequent years (2022 and 2023), as a cost-saving measure, we opted to optimize the process by reducing the number of SNPs from six to one. It is noteworthy that all data analyzed, whether utilizing the complete block, two, or even just one SNP, fit the segregation ratio for one gene (Table 3). These results suggest that in early generations, it is possible to select resistant materials (around 25%) with reliability, leading to the removal of a significant number of lines, thereby reducing costs and facilitating the handling of materials for additional trait evaluations. While we specifically selected SNP8, it is essential to emphasize that any of the six SNPs could serve as a valuable marker in backcrossing or early-generation crossing programs. 

## 4. Materials and Methods

### 4.1. Plant Material

A collection of 61 chickpea accessions consisting of 14 cultivars (ID: 1 to 14) and 47 advanced resistant lines in F_6_/F_7_ (ID: 15 to 61) developed in our breeding program and specifically evaluated for Foc5, was used in the present study (Table 1). Those lines had WR315 and ICCL81001 as sources of resistance. The resistance phenotyping of these lines was conducted in the F_2_ generation in fields naturally infected in Escacena del Campo (Huelva, South of Spain). The assessment was based on visual observations distinguishing between alive and dead plants. The evaluation took into consideration the recessive nature of the resistance, as outlined by Tekeoglu et al. [4]. “Blanco Lechoso” and ILC3279 were used as susceptible controls, whereas “Ituci” and WR315 served as resistant controls. Three resistant genotypes (WR315, ICCL81001, and “Ituci”) and four susceptible genotypes (JG62, ILC2956, ILC3279, and “Blanco Lechoso”) were employed as controls to confirm whether these materials carried the alleles associated with resistance or susceptibility (Table 1).

Additionally, we used 67 inbred lines (ID: 62 to 128) previously evaluated for disease reaction to several Foc races in different studies. All of the inbred lines were supplied by USDA-ARS Washington State University, USA. The genotypes WR315 and P-2245, reported as differential lines to discriminate between resistance and susceptibility to all Foc races [13], were employed as controls for these materials (Table 2).

In an effort to introduce new variability into our breeding program, we developed 68 F_1_ plants derived from multiple crosses between susceptible and resistant Foc5 genotypes, with WR315 as the source of resistance. These F_1_ generated a total of 3634 F_2_ plants that were sown during three different agronomic seasons: 2021 (498 plants), 2022 (1885 plants), and 2023 (1251 plants) (Table 3). This material was used to assess the efficacy of SNP markers in selecting resistant genotypes in F_2_.

### 4.2. DNA Extraction and SNP Genotyping

Genomic DNA was isolated from lyophilized young leaves of one plant per genotype using the E-Z 96 Plant DNA Kit (Omega Bio-Tek, Norcross, GA, USA). The samples were diluted at a concentration of 10 ng/μL.

The panel of six SNPs (Table 4; SNP8; SNP14; SNP24; SNP30; SNP36; and SNP40) developed by Caballo et al. [12], was utilized for genotyping the 128 aforementioned genotypes (Table 1 and Table 2) using iPLEX-Sequenom. The genotyping service was carried out at CEGEN-CNIO (https://www.cnio.es/investigacion-e-innovacion/servicios/unidad-de-genotipado-humano-cegen/, accessed on 29 November 2023).

For the MAS application, the SNPs were employed in our breeding program to genotype the population of 3634 F_2_ plants (Table 3). The genotypes WR315 and ILC3279 were included as controls to identify the allele associated with resistance/susceptibility. Specifically, in 2021, the targeted genotyping with the block of six SNPs was performed using the iPLEX-Sequenom technique. In 2022 and 2023, for enhanced cost-effectiveness, only two SNPs (SNP8 and SNP40) located at the extremes of the targeted region were selected for the development of a TaqMan assay. The genotyping service was carried out at the Genotyping Unit-CEGEN in the Spanish National Cancer Research Centre (CNIO). Primer and probe sequences for each target SNP were synthesized by Thermo Fisher Scientific (Waltham, MA, USA) and are listed in Table 4. The PCR reaction was performed using TaqMan Genotyping Master Mix (Thermo Fisher Scientific, Waltham, MA, USA), utilizing the ABI PRISM 7900HT Sequence Detection System, and subsequently analyzed using the TaqMan Genotyper Software v1.7.1. The cycling protocol comprised an initial cycle at 95 °C for 10 min, followed by 40 cycles at 92 °C for 15 s and 60 °C for 1 h.

### 4.3. Functional Annotation 

The sequences contained in the genomic region delimited by SNP8 and SNP40 (LG2: 23,311,757–24,828,612) were downloaded from the genome browser (Genome Data Viewer) at NCBI. Next, the sequences were submitted to the functional annotation pipeline using the Blast2GO methodology [25] implemented in the OmicsBox platform (BioBam Bioinformatics, Valencia, Spain, v. 2.1.10). For the mapping and annotation, the following configuration settings were used: BLASTP against NCBI non-redundant (nr) protein database, E-value filter ≤ 10^−6^, length cutoff of 33, maximum 5 BLAST hits per sequence, and annotation cutoff of 50. Furthermore, to improve annotation, InterProScan was performed, and results were merged into GO annotation.

## Figures and Tables

**Table 1:** Phenotypic reaction and SNP haplotypes related to Foc5 in a panel of chickpea genotypes (controls, cultivars, and advanced lines).

ID	Material Type	Accessions/Genotypes	Phenotype ^1^	SNP Markers ^2^						Phenotypic Evaluation Reference ^3^
				8	14	24	30	36	40	
	Controls	WR315	R	A	A	A	C	A	A	[13]
		ICCL81001	R	A	A	A	C	A	A	[8]
		“Ituci”	R	A	A	A	C	A	A	Agrovegetal Company
		JG62	S	G	C	G	G	G	T	[13]
		ILC2956	S	G	C	G	G	G	T	[12]
		ILC3279	S	G	C	G	G	G	T	[8]
		“Blanco Lechoso”	S	G	C	G	G	G	T	Local landrace
1	Cultivars	“Italica”	R	A	A	A	C	A	A	Agrovegetal Company
2		“Tarsis”	R	A	A	A	C	A	A	“
3		“Longano”	R	A	A	A	C	A	A	[14]
4		“Surutato-77”	R	A	A	A	C	A	nd	[13]
5		“Fardón”	S	G	C	G	G	G	T	Córdoba breeding program
6		“Juano”	S	G	C	G	G	G	T	“
7		“Pringao”	S	G	C	G	G	G	T	“
8		“Kaveri”	S	G	C	G	G	G	T	“
9		“Veleka”	S	G	C	G	G	G	T	“
10		BT5-7	S	G	C	G	G	G	T	“
11		RR-51	S	G	C	G	G	G	T	“
12		CA2139	S	G	C	G	G	G	T	Local landrace
13		“Macarena”	S	G	C	G	G	G	T	[15]
14		“Breve_Blanco”	S	G	C	G	G	G	T	“
15	Advanced lines	WR 4-p29	R	A	A	A	C	A	A	This study
16		WR 4-p31	R	A	A	A	C	A	A	“
17		WR 4-p80	R	A	A	A	C	A	A	“
18		C13-p7	R	A	A	A	C	A	A	“
19		C13-p15	R	A	A	A	C	A	A	“
20		C27-p8	R	A	A	A	C	A	A	“
21		C39-p28	R	A	A	A	C	A	A	“
22		CAVIR 3A-p62	R	A	A	A	C	A	A	“
23		CAVIR 3A-p65	R	A	A	A	C	A	A	“
24		CAVIR 3B-p10	R	A	A	A	C	A	A	“
25		CAVIR 3B-p11	R	A	A	A	C	A	A	“
26		CV3A 45-1	R	A	A	A	C	A	A	“
27		CV3B 33-1	R	A	A	A	C	A	A	“
28		CV3B 43-1	R	A	A	A	C	A	A	“
29		CV3B 45-1	R	A	A	A	C	A	A	“
30		TK1A	R	A	A	A	C	A	A	“
31		TK2A	R	A	A	A	C	A	A	“
32		TK3	R	A	A	A	C	A	A	“
33		TK3A	R	A	A	A	C	A	A	“
34		TK7A	R	A	A	A	C	A	A	“
35		TK13	R	A	A	A	C	A	A	“
36		TK16	R	A	A	A	C	A	A	“
37		TK18	R	A	A	A	C	A	A	“
38		TK19	R	A	A	A	C	A	A	“
39		TK20	R	A	A	A	C	A	A	“
40		TK21	R	A	A	A	C	A	A	“
41		96-1	R	A	A	A	C	A	A	“
42		105-4	R	A	A	A	C	A	A	“
43		116-1	R	A	A	A	C	A	A	“
44		409-4	R	A	A	A	C	A	A	“
45		409-5	R	A	A	A	C	A	A	“
46		412-1	R	A	A	A	C	A	A	“
47		414-1	R	A	A	A	C	A	A	“
48		414-2	R	A	A	A	C	A	A	“
49		TB13	R	A	A	A	C	A	A	“
50		TB15	R	A	A	A	C	A	A	“
51		TB16	R	A	A	A	C	A	A	“
52		TB18	R	A	A	A	C	A	A	“
53		TB21	R	A	A	A	C	A	A	“
54		TB38	R	A	A	A	C	A	A	“
55		TB41	R	A	A	A	C	A	A	“
56		TB43	R	A	A	A	C	A	A	“
57		TB44	R	A	A	A	C	A	A	“
58		TB46	R	A	A	A	C	A	A	“
59		TB47	R	A	A	A	C	A	A	“
60		TKR14	R	A	A	A	C	A	A	“
61		TKS38	R	A	A	A	C	A	A	“

^1^ R (resistant) and S (susceptible). ^2^ nd (non-data). ^3^ “ Idem.

**Table 2:** Phenotypic reaction and SNP haplotypes related to Foc in a panel of chickpea inbred lines.

ID	Material Type	Accessions/Genotypes	Phenotype ^1^	SNP Markers ^2^						Phenotypic Evaluation Reference ^3^
				8	14	24	30	36	40	
	Resistant haplotype	WR315	R (all races)	A	A	A	C	A	A	[13]
	Susceptible haplotype	P-2245	S (all races)	G	C	G	G	G	T	[13]
62	Inbred lines	JG11	R (nd)	A	A	A	C	A	A	[16]
63		CRIL1_94	R (1,3)	A	A	A	C	A	A	[13]
64		ICCV 92944/W6 48921	R (nd)	A	A	A	C	A	A	[17]
65		ICCV 04512/W6 48936	R (nd)	A	A	A	C	A	A	[18]
66		ICC 2072/W6 25960	R (nd)	A	A	A	C	A	A	ICRISAT Genebank
67		ICC8170	R (nd)	A	A	A	C	A	A	“
68		ICC8182	R (nd)	A	A	A	C	A	A	“
69		ICC8185	R (nd)	A	A	A	C	A	A	“
70		ICC8221	R (nd)	A	A	A	C	A	A	“
71		ICC8222	R (nd)	A	A	A	C	A	A	“
72		ICC8249	R (nd)	A	A	A	C	A	A	“
73		ICC8250	R (nd)	A	A	A	C	A	A	“
74		ICC9499	R (nd)	A	A	A	C	A	A	“
75		ICCV 4/W6 3530	R (1)	A	A	A	C	A	A	[15]
76		ILC99/W6 2905	S	G	C	G	G	G	T	[19]
77		ILC182/W6 22576	S	G	C	G	G	G	T	“
78		ILC200/W6 10143	S	G	C	G	G	G	T	“
79		ILC187/W6 14952	S	G	C	G	G	G	T	“
80		ILC189/W6 14953	S	G	C	G	G	G	T	“
81		ILC191/W6 22577	S	G	C	G	G	G	T	“
82		ILC194/W6 22578	S	G	C	G	G	G	T	“
83		ILC200/W6 22579	S	G	C	G	G	G	T	“
84		ILC215/W6 22580	S	G	C	G	G	G	T	“
85		ILC482/W6 22582	S	G	C	G	G	G	T	“
86		ILC484/W6 22583	S	G	C	G	G	G	T	“
87		Dwelley (PI 598079)	R (1,2)	G	C	G	G	G	T	[13]
88		ICC8159	R (nd)	G	C	G	G	G	T	ICRISAT Genebank
89		ICC8165	R (nd)	G	C	G	G	G	T	“
90		ICC8200	R (nd)	G	C	G	G	G	T	“
91		ICC8204	R (nd)	G	C	G	G	G	T	“
92		ICC9490	R (nd)	G	C	G	G	G	T	“
93		ICC9496	R (nd)	G	C	G	G	G	T	“
94		ICC14449	R (nd)	G	C	G	G	G	T	“
95		SAKI9516	R (nd)	A	A	A	C	A	T	[16]
96		ICCV 10/PI 578283	R (nd)	G	C	A	C	A	A	“
97		ICC2065/W6 25959	S	A	A	A	C	A	A	ICRISAT Genebank
98		ICC8384/W6 26034	S	A	A	A	C	A	A	“
99		ILC249/W6 22581	S	A/G	C	G	G/C	G	T	[19]
100		C 214/PI 374097	I (1)	A	A	A	C	A	A	[20]
101		ICCV 04514/W6 48937	I (nd)	A	A	A	C	A	A	[18]
102		ICC6465	I (nd)	A	A	A	C	A	A	ICRISAT Genebank
103		ICC6628	I (nd)	A	A	A	C	A	A	“
104		ICC8184	I (nd)	A	A	A	C	A	A	“
105		ICC8224	I (nd)	A	A	A	C	A	A	“
106		ICC8226	I (nd)	A	A	A	C	A	A	“
107		ICC8228	I (nd)	A	A	A	C	A	A	“
108		ICC8239	I (nd)	A	A	A	C	A	A	“
109		ICC8240	I (nd)	A	A	A	C	A	A	“
110		ICC8241	I (nd)	A	A	A	C	A	A	“
111		ICC8243	I (nd)	A	A	A	C	A	A	“
112		ICC8245	I (nd)	A	A	A	C	A	A	“
113		ICC8251	I (nd)	A	A	A	C	A	A	“
114		ICC8252	I (nd)	A	A	A	C	A	A	“
115		ICC8253	I (nd)	A	A	A	C	A	A	“
116		ICC9488	I (nd)	A	A	A	C	A	A	“
117		ILC136/W6 2844	I (nd)	G	C	G	G	G	T	[19]
118		ILC213	I (nd)	G	C	G	G	G	T	ICRISAT Genebank
119		ILC254	I (nd)	G	C	G	G	G	T	“
120		ICC8190	I (nd)	G	C	G	G	G	T	“
121		ICC8205	I (nd)	G	C	G	G	G	T	“
122		ICC8206	I (nd)	G	C	G	G	G	T	“
123		ICC8207	I (nd)	G	C	G	G	G	T	“
124		ICC9491	I (nd)	G	C	G	G	G	T	“
125		ICC9495	I (nd)	G	C	G	G	G	T	“
126		ICC9501	I (nd)	G	C	G	G	G	T	“
127		ICC9515	I (nd)	G	C	G	G	G	T	“
128		ICC5704	I (nd)	G	C	G	G	G	A	“

^1^ R (resistant), S (susceptible), I (intermediate), between brackets races reported, nd (non-data). ^2^ Gray shaded lines correspond to genotypes with non-coincident phenotype and genotype. ^3^ “ Idem. ICRISAT Genebank, Patancheru, Telangana, India. 2017/supplied by USDA-ARS.

**Table 3:** The number of F_1_ and F_2_ derived from different crosses realized in different years, and their segregation when genotyped either with the complete SNP block or just two or a single SNP.

				Markers for Genotyping	Segregation ^2^		
Cross	Year	F_1_	F_2_ Analyzed		R	H	S
TK-18 x LC182	2021	6	154	SNP block ^1^	39	71	44
TK-18 x LC187		6	137		30	75	32
TK-18 x ILC5921		9	182		52	87	43
412-1 x ILC182		1	10		5	3	2
412-1 x ILC187		2	15		3	9	3
**Total**			**498**		**129**	**245**	**124**
TK-18 x LC187	2022	1	66	SNP8/SNP40	16	36	14
TK-18 x ILC5921		4	397		88	219	90
414-2 x ILC182		1	122		30	70	22
414-2 x ILC187		4	441		117	226	98
414-2 x LC5921		2	207		62	100	45
Tarsis x ILC182		1	92		25	38	29
96-1 x ILC182		2	215		53	116	46
96-1 x ILC187		1	132		33	67	32
96-1 x ILC5921		2	213		62	97	54
**Total**			**1885**		**486**	**969**	**430**
412-1 x ILC182	2023	3	209	SNP8	48	111	50
412-1 x ILC187		2	91		20	52	19
412-1 x LC5921		4	123		33	57	33
414-2 x ILC182		1	29		7	15	7
ILC5921 × 414-2		1	36		10	15	11
Tarsis x ILC187		4	141		37	79	25
Tarsis x ILC5921		3	163		40	77	46
Ituci x ILC182		1	64		13	35	16
Ituci x ILC187		4	220		65	110	45
Ituci x ILC5921		2	128		33	66	29
96-1 x ILC187		1	47		8	21	18
**Total**			**1251**		**314**	**638**	**299**

^1^ SNP block = SNP8, SNP14, SNP24, SNP30, SNP36, and SNP40. ^2^ R (resistant), H (heterozygous), S (susceptible).

**Table 4:** Panel of six SNPs developed by Caballo et al. [12].

SNP (ID)	Physical Position in Ca2 (bp)	Haplotype Ref Genome	Haplotype Resist Foc5	Haplotype Suscept Foc5	Primers Iplex Sequenom	Size Amp. (bp)	Primers/Probes Taqman	Size Amp. (bp)
SNP8	23,311,757	G	A	G	L:ACGTTGGATGAGGTAGACTGATAGTGGTCG	112	L: GTGAGTTTTGTGGAGTTGAGTTAGACT	82
					R:ACGTTGGATGGTGAGTTTTGTGGAGTTGAG		R: TGGTCGGCCCAATAGGTTTT	
					Extent: AGACTCAATTCAAATTGATAGAATC		Reporter1-Vic:CAAATTGATAGAATCGGAGCAT	
							Reporter2-Fam:AAATTGATAGAATCAGAGCAT	
SNP14	23,402,082	C	A	C	L:ACGTTGGATGGCAGAAGTTTGCTTGAAGAG	108		
					R:ACGTTGGATGTTGGAAGATGAAGGCAAAAG			
					Extent:CTTTACAAATCTACCAACACTT			
SNP24	24,033,600	G	A	G	L:ACGTTGGATGATATACGACGGAAGCGAAGG	120		
					R:ACGTTGGATGTACCAATGCCCCCTTCTTTG			
					Extent:TGGTAAGATTGGACATAATCA			
SNP30	24,515,122	G	C	G	L:ACGTTGGATGGACCCCGATTAGTGTGAAAC	100		
					R:ACGTTGGATGTGACTCATGCTCTGGTTATC			
					Extent:CTCTGGTTATCACCAAATT			
SNP36	24,686,576	G	A	G	L:ACGTTGGATGTTGATCATTCGCTCGAATT	98		
					R:ACGTTGGATGTCAACAAGTTTTATACCGC			
					Extent:CCAAGTTTTATACCGCAAATAAAAA			
SNP40	24,828,612	T	A	T	L:ACGTTGGATGCACATCTCTCTCACATGCTG	112	L:GGCTACATCTTTGTGTTGTTATCTACTGT	84
					R:ACGTTGGATGTCGAGACATGTAATGGCTAC		R:CAACACATCTCTCTCACATGCTGAA	
					Extent:TACTGTCTTTTTGTAGGTTGATGT		Reporter1-Vic:ATGACCCAACATCAAC	
							Reporter2-Fam:AATGACCCTACATCAAC	

## Data Availability

The data presented in this study are available within the article.

## Supplementary Materials

The following supporting information can be downloaded at: https://www.mdpi.com/article/10.3390/plants13030436/s1. Table S1: Functional annotation data.
